# A Study of Adjacent Intersection Correlation Based on Temporal Graph Attention Network

**DOI:** 10.3390/e26050390

**Published:** 2024-04-30

**Authors:** Pengcheng Li, Baotian Dong, Sixian Li

**Affiliations:** School of Traffic and Transportation, Beijing Jiaotong University, Beijing 100044, China; bjtulpc@163.com (P.L.); 20114056@bjtu.edu.cn (S.L.)

**Keywords:** machine learning, intersection correlation degree, intersection state classification, temporal graph attention network, information gain

## Abstract

Traffic state classification and relevance calculation at intersections are both difficult problems in traffic control. In this paper, we propose an intersection relevance model based on a temporal graph attention network, which can solve the above two problems at the same time. First, the intersection features and interaction time of the intersections are regarded as input quantities together with the initial labels of the traffic data. Then, they are inputted into the temporal graph attention (TGAT) model to obtain the classification accuracy of the target intersections in four states—free, stable, slow moving, and congested—and the obtained neighbouring intersection weights are used as the correlation between the intersections. Finally, it is validated by VISSIM simulation experiments. In terms of classification accuracy, the TGAT model has a higher classification accuracy than the three traditional classification models and can cope well with the uneven distribution of the number of samples. The information gain algorithm from the information entropy theory was used to derive the average delay as the most influential factor on intersection status. The correlation from the TGAT model positively correlates with traffic flow, making it interpretable. Using this correlation to control the division of subareas improves the road network’s operational efficiency more than the traditional correlation model does. This demonstrates the effectiveness of the TGAT model’s correlation.

## 1. Introduction

Intersections are key areas for vehicle convergence and evacuation [1]. To improve the efficiency of vehicle traffic and effectively reduce traffic congestion, it is necessary to carry out traffic management on intersections and formulate a reasonable traffic signal control scheme.

There are two important concepts in intersection management: intersection traffic status and intersection correlation degree. On the one hand, the real-time traffic state of an intersection can reflect the suitability of the current traffic flow and the signal control scheme, which provides a basis for finding the key points of traffic congestion; on the other hand, the correlation between adjacent intersections can reflect the necessity for coordinated control [2]. The implementation of more highly correlated and linked intersections will reduce stopping delays and improve the level of service of the entire roadway network.

In terms of the traffic state, in the existing research, it is mainly transformed into a clustering problem through the extraction of the feature vectors of the traffic state from traffic data, such as speed, flow rate, time occupancy, etc., and then artificial intelligence algorithms are used to solve the problem, such as the spectral clustering algorithm [3], k-means algorithm [4], DBSCAN algorithm [5], etc. The shortcomings of the existing research are as follows: (1) The object of many studies is the traffic state of road sections, not the traffic state of intersections. The difference is that the feature dimension of the road section traffic data is generally low, and the traffic flow generates new feature parameters such as queue length, number of stops, flow ratio, etc., at signal-controlled intersections. In other words, the original road section features cannot comprehensively describe the traffic state of the intersection. (2) The performance of traditional machine learning algorithms decreases with a significant increase in feature dimensions and data volume, making it difficult to apply them to large-scale road networks.

In terms of the intersection correlation degree, the existing research mainly aims to establish the corresponding mathematical function and calculate the intersection correlation degree quantitatively according to the patterns of the change in traffic data between adjacent intersections. The Whitson model [6] is a typical representative of the correlation function that takes into account many parameters between neighbouring intersections, such as the average travel time, maximum directional traffic flow, and total traffic flow. If the correlation degree is closer to 1, the more suitable the two intersections are for coordinated control. Many scholars have improved the Whitson model, such as by adding correction parameters and expanding or creating brand-new correlation formulas from other aspects such as the degree of traffic dispersion and intersection spacing. The shortcomings of the existing research are as follows: (1) Some specific parameters in the intersection correlation degree model need to be manually set according to experience, such as the weight coefficient, which makes the model itself subjective. (2) Some parameters, such as the number of vehicle transfers and saturation dissimilarity, need to be calculated by combining a variety of inputs, during which errors are cumulative and constantly deviate from the actual values. Most importantly, these mathematical models lack a self-adjustment mechanism, which obviously affects the reliability of the correlation.

In order to solve the above problems, this paper uses graph theory to abstract intersections as nodes in the graph and road sections as edges in the graph, so as to transform the intersection state classification problem into the classification problem of nodes and the intersection relevance into the relevance of nodes. An intersection correlation degree model based on temporal graph attention is established to solve the intersection traffic state classification problem and the intersection correlation problem at the same time. Specifically, we establish a temporal graph attention (TGAT)-based intersection correlation degree model, where the data source is the VISSIM simulation results. The inputs are the traffic characteristics of each intersection, the interaction time of the intersections, and the initial labels of the intersections. The outputs are the node embeddings of the target node and its neighbour node weights at any moment. Then, the node states are inferred from the node embeddings, and the neighbour node weights are aggregated as the correlation of the adjacent intersections. Finally, the classification accuracy of the TGAT model is compared with three traditional classification models. Meanwhile, the relationship between the correlation degree and roadway flow was quantitatively analysed, the roadway network nodes were divided into different control subareas based on the correlation degree, and the validity of the correlation degree was verified by comparing the operational efficiency of the whole roadway network.

The rest of this paper is organized as follows: Section 2 describes the existing relevant research methods for traffic state classification and intersection correlation. Section 3 presents the TGAT-based intersection correlation degree model. Section 4 shows the simulation experiments based on real data and the validation of the classification accuracy and correlation of the TGAT model. Section 5 discusses the results of the experiment. Section 6 summarizes the full work and provides an outlook on future research directions.

## 2. Related Works

### 2.1. Research on Classification of Traffic Flow States

The accurate classification of traffic states is the basis for the development of traffic control schemes, and scholars have carried out a lot of research on this topic. Wang et al. [7] proposed a traffic state classification model based on a feature graph and deep learning, converted the traffic state feature vector into a feature graph, and established a traffic state classification model based on the CoAtNet algorithm, which was verified by vehicle trajectory data in Shanghai city. Zhang et al. [8] selected volume, speed, and occupancy as inputs, quantitatively analysed the influence of different traffic flow parameters on traffic state classification, and proposed a weighted fuzzy c-means clustering method for traffic classification, which has a better classification accuracy than that of the traditional algorithm. Tu et al. [9] chose vehicle acceleration, angular acceleration, and GPS speed data to establish a deep belief network model that proved that acceleration and angular acceleration data can increase the accuracy of traffic flow classification significantly. Hu et al. [10] conducted experiments using measured data from floating vehicles, set thresholds for the different phases of traffic flow at intersections, and combined various data such as instant direction, instant speed, GPS position, time, etc., to identify the traffic states. Yuan et al. [11] selected various variables such as the average speed, stopping times, minimum speed, etc., and compared the performance of various models such as Convolutional Neural Networks (CNNs), gradient boosting decision trees, and long short-term memory neural networks, etc. Yang et al. [12] combined five typical daily traffic state change clusters with different patterns and classified the traffic state using a spectral clustering algorithm.

### 2.2. Research on Intersection Correlation Degrees

Shen et al. [13] analysed the influences of the segment distance, traffic flow density, and signal cycle time on the correlation degree of two adjacent intersections and proposed a fuzzy calculation method based on a hierarchical structure to estimate the correlation degree. Hu et al. [14] selected five variables between two intersections, including the flow, signal timing correlation degree, travel time, queue length, and delay, and then built separate correlation models and combined them into a composite correlation, which was used as a basis for the coordinated control of arterial roads, and improved the operational efficiency of the arterial road network. Bie et al. [15] proposed the correlation degree index (CI) to represent the correlation degree of intersections by taking into account the cycle, flow, and road length and used numerical experiments; the calculation was complicated. Ke et al. [16] proposed a correlation degree calculation method based on the distance and flow between adjacent intersections, combined with the Newman algorithm and an automatic license plate recognition algorithm, conducted subarea division experiments under large-scale road networks, and verified their effectiveness. Pang et al. [17] proposed a coupling model of adjacent intersections of highways and established a density-based traffic transfer equation for evaluating the coupling strength of adjacent intersections.

### 2.3. Research on Graph Neural Networks

A graph neural network is a deep learning method based on graph structure. Kipf et al. [18] presented a scalable approach for semi-supervised learning on graph-structured data, which improved the accuracy of classification models based on graph structures. On this basis, Velickovi et al. [19] presented graph attention networks (GAT), without requiring any kind of costly matrix operation or dependence on knowing the graph structure upfront, which laid the foundation for subsequent research on various types of GAT models.

The road network is a natural graph structure, and in recent years, many scholars have used graph neural networks to solve problems in traffic road networks. The spatial correlation in the traffic network is captured using graph neural networks, and the temporal correlation in the time series is captured using time series models. For example, Wang et al. [20] designed a graph neural framework with a GAT and LSTM network (MetaSTGAT) to obtain spatial and temporal information. Fang et al. [21] proposed an end-to-end neural framework named ConSTGAT to estimate travel time. Zhang et al. [22] proposed a spatial–temporal convolutional graph attention network for traffic flow prediction. Wu et al. [23] proposed a novel neural network framework called DynSTGAT to solve the signal control problem.

In 2020, Xu et al. [24] proposed the temporal graph attention (TGAT) model at the top deep learning conference, the International Conference on Learning Representations (ICLR). They encoded time and used self-attention mechanisms to deal with continuous time, and their experimental analysis demonstrated the effectiveness of their approach for capturing temporal feature signals in terms of both node and topological features on temporal graphs. It was considered one of the most important research results in the field of graph neural networks in recent years. On the one hand, as a classification model, compared with traditional machine learning models, the performance of TGAT is less restricted by input dimensions and has been shown to be applicable to multi-dimensional datasets such as Reddit, Wikipedia, Industrial, etc., and its classification accuracy is generally better than that of traditional machine learning models, so it can be used to widely select traffic features at intersections and more comprehensively describe the traffic status of intersections. On the other hand, since TGAT contains a self-attention mechanism, the self-attention value can be used as the node’s relevance; compared with traditional relevance models [13,14,15,16,17], TGAT does not require the additional input of artificial parameters; and compared with static graphs such as GraphSage (Graph Sample and Aggregate), GAT, and so on, it can continuously adjust the weight coefficients of the neighbouring nodes in training, and it can more clearly show the influence of nodes on the current road network structure at different moments and obtain the dynamic correlation of intersections.

However, the TGAT model cannot be used directly because it only considers the time factor in the process of capturing neighbouring nodes, which will lead to two intersections that are far away from each other being incorrectly defined as highly similar because of their similar interaction times. Therefore, in this paper, we propose an intersection correlation degree model based on the TGAT, which redefines the interaction time of intersections and improves the capture process of the nearest neighbour nodes in the model to make it applicable to road network nodes, which can simultaneously solve the two problems of intersection traffic state classification and intersection correlation. We established a simulation environment based on measured traffic data and verified the classification accuracy and correlation validity of the model through simulation experiments.

## 3. Materials and Methods

**Figure 1:**
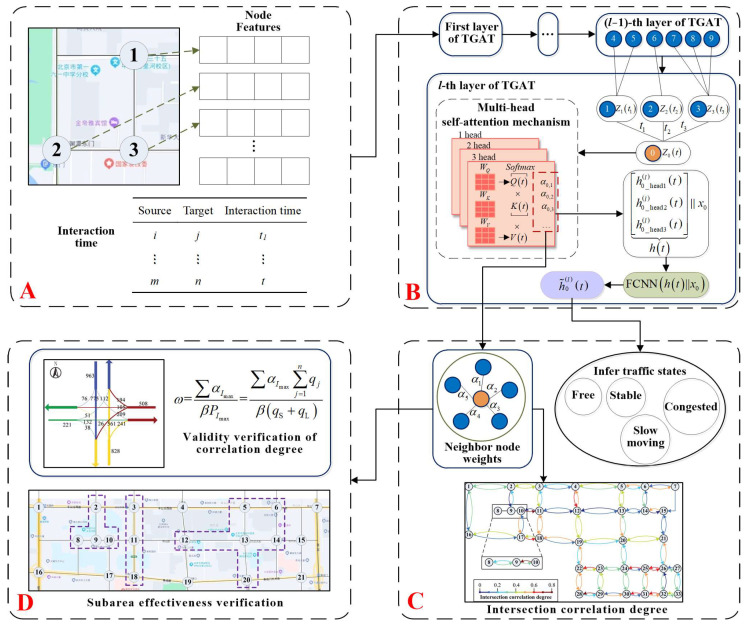
Overall framework. (**A**) Input data includes node characteristics and interaction times; (**B**) The model composed of multi-layer TGAT; (**C**) Translate the output of the TGAT model into neighbor node weights and node traffic states; (**D**) Validation of correlation by parameter “ω” and subarea experiment.

For ease of description, intersections are collectively referred to as nodes in the following. Figure 1 shows the framework of this paper, which is divided into four parts.

Figure 1A is to transform the traffic characteristics of the nodes into a feature matrix and determine the node interaction times. Figure 1B is to build the TGAT-based intersection correlation model in layer l. After inputting the node feature matrix and the time of node interaction, the neighbouring node weights of each node in layer *l* at different moments are obtained through layer-by-layer iteration, and the embedding value of the target node is obtained. Figure 1C is to superimpose the neighbour node weights into the correlation degree of the nodes and infer the state labels of the nodes based on the node embedding. Figure 1D verifies the interpretability of the correlation in terms of flows and uses the correlation of the different models to partition subareas in order to verify the effectiveness of the correlation in improving the operational efficiency of the road network.

### 3.1. TGAT-Based Intersection Correlation Model

The inputs to build the TGAT-based intersection correlation model are intersection feature matrix F, intersection interaction time matrix T, and intersection initial labelling matrix L, and the outputs are the traffic states of the target intersection and the correlation of adjacent intersections at different moments.

#### 3.1.1. Selection of Inputs

(1) Intersection feature matrix F

The features of an intersection can be divided into traffic flow features and physical features. In this paper, the traffic flow features are collected by node detectors and data point detectors in the VISSIM simulation environment; the physical features are obtained by field traffic survey. The specific parameters are shown in Table 1.

Since all the intersections in the experimental area are 3–4 directional inlets, the features of each intersection are set according to the 4 inlets—east, west, south, and north—and all the data for the missing directions are set to 0. Thus, the feature matrix for the east inlet of an intersection can be expressed as the following:

$$f_{E}=\left[D_{\mathrm{ave}E},T_{\mathrm{stopd}E},N_{\mathrm{stops}E},N_{\mathrm{veh}E},L_{\mathrm{queue}E},C_{E},T_{\mathrm{sg}E},T_{\mathrm{lg}E}\right]$$

Then, the feature matrix of an intersection can be expressed as $F=\left[f_{E},f_{W},f_{S},f_{N},T\right]$, where the dimension is 33.

(2) Interaction time matrix T

**Figure 2:**
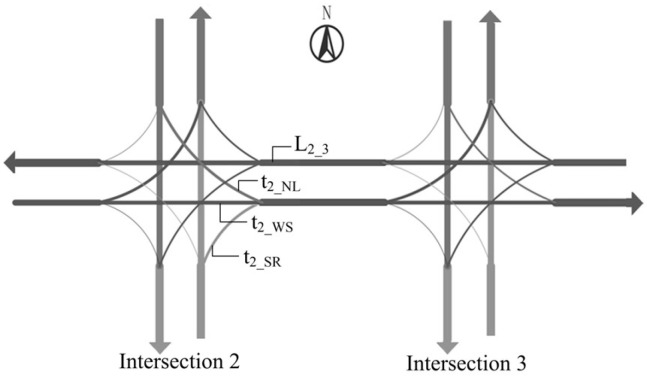
Interaction path schematic.

In the TGAT model, it is necessary to determine the time at which information interaction occurs at two intersections. We use the example of intersection 2 and intersection 3 in part A of Figure 1 to define the interaction time. It is assumed that the phases of both intersections are [east–west straight, east–west left turn, north–south straight, north–south left turn]. As shown in Figure 2, there are 3 time periods ($t_{2\_NL}$, $t_{2\_WS}$, $t_{2\_SR}$) in a cycle when vehicles at intersection 2 can drive towards intersection 3, i.e., there are 3 ways of interacting with intersection 3:

$t_{2\_NL}$ denotes the left-turn green time at the north inlet of Intersection 2, $t_{2\_WS}$ denotes the straight green time at the west inlet of intersection 2, and $t_{2\_SR}$ denotes the right-turn time at the south inlet of intersection 2. We only need to consider the first two scenarios because right-turning traffic is not controlled by traffic signals.

Traffic in the actual driving process is discrete, which makes it difficult to form a unified arrival time. So, we choose the median of the effective green time as the start time. Then, the distance of the road section ($L_{2-3}$) and average speed in the simulation (${\bar{V}}_{2-3}$) are used to calculate the interaction times $T_{2W-3}$ and $T_{2S-3}$.

(1) $$T_{2N-3}=\frac{t_{2\_NL}}{2}+\frac{L_{2-3}}{{\bar{V}}_{2-3}}$$

(2) $$T_{2W-3}=\frac{t_{2\_WS}}{2}+\frac{L_{2-3}}{{\bar{V}}_{2-3}}$$

Assuming that the west inlet of intersection 2 starts to release at moment x, the two interaction times of intersections 2 and 3 in the time matrix T are $\left[x+T_{2W-3},x+t_{2\_\mathrm{WS}}+t_{2\_\mathrm{WL}}+t_{2\_\mathrm{NS}}+3t_{y}+T_{2N-3}\right]$, where $t_{y}$ is the yellow light time.

(3) Label matrix L

The clustering algorithm (Clustering by Fast Search and Find of Density Peaks, DPC) proposed in the literature [25] is used to divide the feature matrix F into 4 classes (free, stable, slow moving, and congested) based on the mean value of $L_{\mathrm{queue}}$ in each class.

#### 3.1.2. TGAT Network Structure

Part B in Figure 1 is a schematic diagram of the *l*-layer TGAT network, including interaction time, node characteristics, and the multi-head attention mechanism. Each layer is a local aggregation operator, whose inputs are the node embedding in the previous layer and the time difference between the two layers. The output is the target node embedding of the current layer. Assuming that nodes 1–3 in layer *l* have each interacted with the target node 0 once, and the interaction times $t_{1}-t_{3}$ are all earlier than the moment t, the aggregation process of the nodes in layer l is as follows:

(1) Find adjacent nodes of target node 0.

TGAT is only concerned with interactions that occur before time *t* of the target node, but in this paper, adjacent nodes should also consider the positional relationship. Studies in the literature [15] have shown that two intersections are often considered unrelated when the intersection distance exceeds 1 km. Assume that the interaction time between the other nodes and node 0 is $t_{0\_i}$ and the distance is $L_{0\_i}$; then, the adjacent nodes of node 0 in this paper are the following:

(3) $$N_{0}=\left(N_{i}\left|\left(t_{0\_i}<t\right)\right.\right)\cap \left(N_{i}\left|\left(L_{0\_i}<1\;\mathrm{km}\right)\right.\right)$$

The symbol “|” denotes a condition.

(2) Splicing node features

In layer *l*, the feature $Z_{i}\left(t_{j}\right)$ of the ith adjacent node can be expressed as the following:

(4) $$Z_{i}\left(t_{j}\right)={\tilde{h}}_{i}^{\left(l-1\right)}\left(t_{j}\right)||\Phi \left(t-t_{j}\right)$$

The symbol “||” represents concatenation, a common data processing method used in the field of graph theory. The formulae include the node embedding in the previous TGAT layer and the time difference function. ${\tilde{h}}_{i}^{\left(l-1\right)}\left(t_{j}\right)$ denotes the node embedding of node *i* in layer l−1. $\Phi \left(t-t_{j}\right)$ denotes the time difference function between node *i* and the target node at time $t_{j}$, which can be derived according to Bochner’s theorem:

(5) $$\Phi_{d}\left(t\right)=\sqrt{\frac{1}{d}}\left[\cos\left(\omega_{1}t\right),\sin\left(\omega_{1}t\right),\ldots ,\cos\left(\omega_{d}t\right),\sin\left(\omega_{d}t\right)\right]$$

where $\omega_{m}=\left[\omega_{1},\ldots ,\omega_{d}\right]$ is the parameter to be learnt.

(3) Aggregation of target node information

The aggregation of target node information is performed with inputs as the set of target node features and adjacent node features, i.e.,

(6) $$Z\left(t\right)={\left[{\tilde{h}}_{0}^{\left(l-1\right)}\left(t\right)||\Phi_{d}\left(0\right),{\tilde{h}}_{1}^{\left(l-1\right)}\left(t_{1}\right)||\Phi_{d}\left(t-t_{1}\right),\ldots ,{\tilde{h}}_{3}^{\left(l-1\right)}\left(t_{3}\right)||\Phi_{d}\left(t-t_{3}\right)\right]}^{T}$$

where ${\left[Z(t)\right]}_{0}$ is the target node feature and the time difference between node 0 and itself is 0, so its time difference function is $\Phi_{d}\left(0\right)$; ${\left[Z(t)\right]}_{1:3}$ denotes the adjacent node feature.

The aggregated query vectors, key vectors, and value vectors are then obtained using a linear transformation utilising the three weight matrices $W_{Q}$, $W_{K}$, and $W_{V}$ in self-attention, which are used to capture the interactions between the temporal encoding and the node features:

(7) $$Q\left(t\right)={\left[Z(t)\right]}_{0}W_{Q},\;K\left(t\right)={\left[Z(t)\right]}_{1:3}W_{K},\;V\left(t\right)={\left[Z(t)\right]}_{1:3}W_{V}$$

Finally, the attention value is calculated:

(8) $$\mathrm{Attention}\left(Q,K,V\right)=\mathrm{softmax}\left(\frac{QK^{T}}{\sqrt{d}}\right)V$$

In the self-attention mechanism of TGAT, the associativity of adjacent nodes is different from GAT, which used the linear transformation followed by the dot-product to capture pair wise interactions of the hidden factors between entities and the time embeddings. The attention weight $\alpha_{ij}$ between node *i* and node *j* is calculated as the following:

(9) $$\alpha_{ij}(t)=\frac{\exp\left({\left(\left[{\tilde{h}}_{i}\left(t_{i}\right)||\Phi_{d}\left(t-t_{i}\right)\right]W_{Q}\right)}^{T}\left(\left[{\tilde{h}}_{j}\left(t_{j}\right)||\Phi_{d}\left(t-t_{i}\right)\right]W_{K}\right)\right)}{\sum_{k\in N(i;t)}\exp\left({\left(\left[{\tilde{h}}_{i}\left(t_{i}\right)||\Phi_{d}\left(t-t_{i}\right)\right]W_{Q}\right)}^{T}\left(\left[{\tilde{h}}_{k}\left(t_{k}\right)||\Phi_{d}\left(t-t_{k}\right)\right]W_{K}\right)\right)}$$

In this paper, the 3-head attention mechanism is used for aggregation, and the three neighbour weight matrices $\alpha_{0\_\mathrm{headi}}=\left[\alpha_{01\_i},\ldots ,\alpha_{0n\_i}\right]$ obtained from node 0 are averaged to be the final correlation between the target node and its neighbours, where *n* is the number of neighbour nodes in the TGAT parameter.

(4) Fully Connected Neural Network

The three attention values obtained are merged into $h\left(t\right)$ and spliced with $x_{0}$, where $x_{0}={\tilde{h}}_{0}^{\left(l-1\right)}\left(t\right)$ is the node embedding of the 0 node at layer l−1. After the FCNN fully connected layer, the final output of layer *l* is obtained, which is the node embedding of node 0 at moment *t*. Based on the probability of the target node belonging to each type of state inferred from ${\tilde{h}}_{0}^{\left(l\right)}\left(t\right)$ and combined with the initial labelling matrix, the cross entropy loss function (CrossEntropyLoss [26]) is computed.

(10) $$Loss=-\sum_{i=1}^{K}y_{i}\log\left(p_{i}\right)$$

where *K* is the number of classifications and $y_{i}$ is the real label in matrix *L*; if the category is *i*, $y_{i}=1$, otherwise $y_{i}=0$. $p_{i}$ is the probability of *i*.

The model parameters are then updated by error back propagation until the training termination condition is reached.

(5) The state label of the target node inferred from the last training will be used as the final traffic state of the target intersection, and the mean value of $\alpha_{0\_\mathrm{headi}}$ is the weight of the adjacent intersection.

### 3.2. Information Gain

The TGAT model does not inherently discern the significance of each feature in categorizing data. Therefore, this study employs the information gain algorithm in the information entropy theory to pinpoint features that substantially influence category distinctions. Information gain measures the degree to which the presence of feature X decreases the uncertainty surrounding the classification of category Y.

The information gain of feature A on dataset D($g\left(D,A\right)$) is defined as the difference between the empirical entropy of set D($H\left(D\right)$) and the conditional empirical entropy ($H\left(D\left|A\right.\right)$) given the condition of feature A.

Assuming the dataset is D, the number of samples is $\left|D\right|$, the number of categories is K, and $C_{k}$ is the number of samples belonging to category $C_{k}$, where k=1,2,…,K. Assume that feature A has n different values $\left[a_{1},a_{2},\ldots ,a_{i},\ldots ,a_{n}\right]$, and divide D into n subsets based on the values of $a_{i}$, i.e., $\sum_{k=1}^{K}\left|D_{i}\right|=\left|D\right|$. Set the sample collection in subset $D_{i}$ that belongs to category $C_{k}$ as $D_{ik}$, i.e., $D_{ik}=D_{i}\cap C_{k}$.

The steps of the information gain algorithm are as follows:

(1) Calculate the empirical entropy of the dataset D.

(11) $$H\left(D\right)=-\sum_{k=1}^{K}\frac{C_{k}}{D}\log_{2}\frac{\left|C_{k}\right|}{\left|D\right|}$$

(2) Calculate the empirical conditional entropy of feature A for dataset D.

(12) $$H\left(D\left|A\right.\right)=\sum_{i=1}^{n}\frac{\left|D_{i}\right|}{\left|D\right|}H\left(D_{i}\right)$$

(3) Calculate the information gain.

(13) $$g\left(D,A\right)=H\left(D\right)-H\left(D\left|A\right.\right)$$

## 4. Results

Based on actual traffic survey data, we have established a corresponding Vissim simulation environment to validate the classification accuracy and connectivity effectiveness of the TGAT model through simulation experiments.

### 4.1. Experimental Parameters

**Figure 3:**
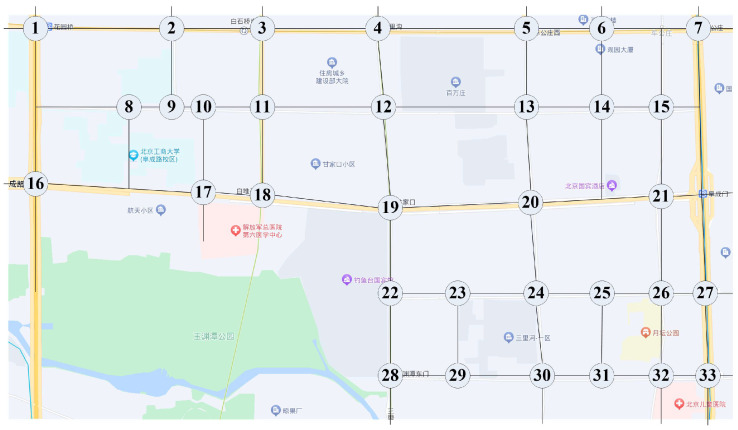
Intersection number schematic.

The traffic volume survey data of a regional road network in Haidian District, Beijing, from 14:00 to 15:00 on a weekday in 2019 were selected for the study. The data included the traffic flow in each direction and traffic signal cycle of each intersection. The road network including 33 signal-controlled intersections is shown in Figure 3.

We set up the VISSIM simulation environment: the maximum simulation time is 6300 s, the data acquisition time is from 1800 to 6300 s, and the interval of the detector’s data acquisition is 300 s. Each inlet on the periphery of the road network increases the traffic volume according to its capacity every 1500 s to simulate the different traffic states of the intersection in different periods.

Three common classification algorithms are selected to verify the classification accuracy of the TGAT model. The first is GWO-MLP [27]: Grey Wolf Optimization (GWO) is used to optimize a Multi-Layer Perceptron (MLP), in which the particle position information of GWO is set as the initial weight and bias in MLP; the second is long short-term memory (LSTM) [28]; and the third is Support Vector Machine (SVM) [29]. The specific parameter settings are shown in Table 2.

### 4.2. Intersection State Identification Results

**Figure 4:**
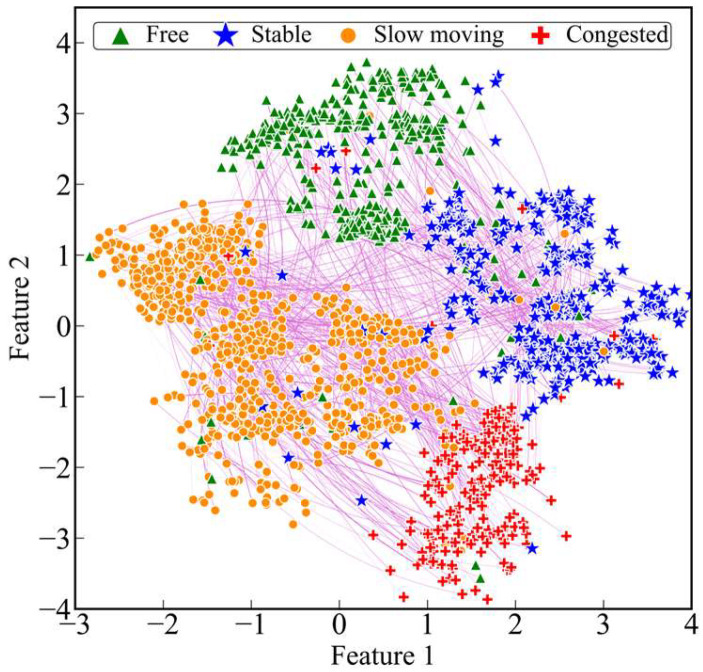
Node embedding visualization results.

The dimension of the target node embedding is consistent with the input volume feature matrix F of TGAT, which cannot be displayed directly. So, the t-SNE algorithm proposed in the literature [30] is used for dimensionality reduction. The results of the dimensionality reduction are shown in Figure 4. 

Due to the small traffic flow of the road network at the beginning of the simulation, the distribution of intersection state labels is not balanced, so the 30th to 60th interaction information points of each intersection are selected to be displayed. The data involved in the dimensionality reduction are the target node and neighbour node embeddings from the final output of TGAT, which are reduced to a two-dimensional space.

Points of different shapes in Figure 4 correspond to different labels, and the thickness of the lines represents the magnitude of the weights among the nodes. It can be seen that all the points are roughly divided into four clusters, and the boundaries between the clusters are relatively clear, indicating that TGAT can not only identify the traffic state of the target intersection at different moments but also quantitatively describe its correlation value with the neighbouring intersections.

**Figure 5:**
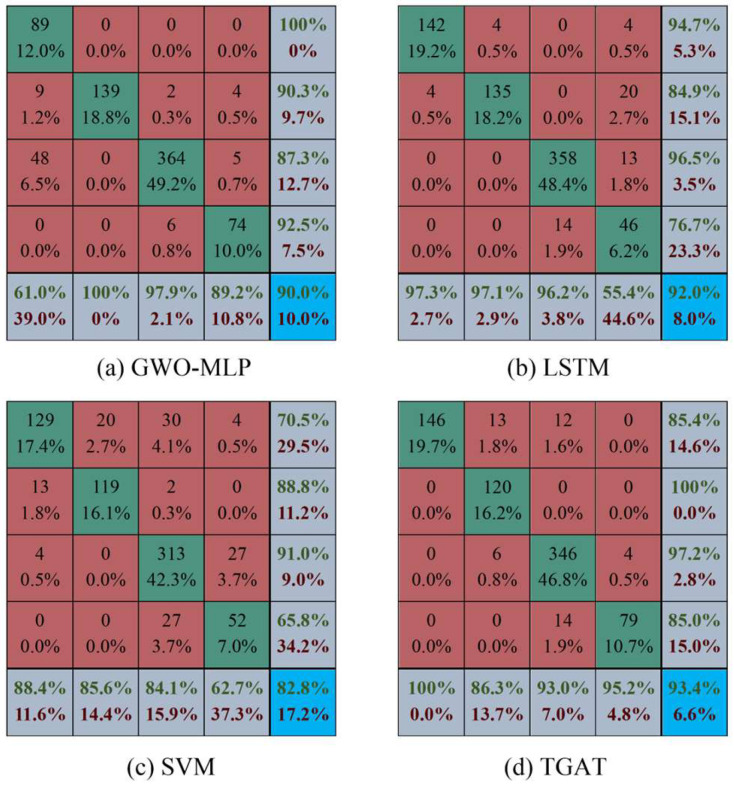
Confusion matrix for each algorithm test set.

The total number of samples during the simulation time period is 4933, representing the number of times each node was interacted with. The division ratio of training, validation, and testing is [0.7, 0.15, 0.15], and the test set confusion matrix for the four algorithms is shown in Figure 5.

In Figure 5, the four traffic states of free, stable, slow moving, and congested are labelled as labels 1 to 4. The integers within the diagonal of the confusion matrix are the number of correctly predicted labels in each category, and the columns indicate the prediction of the current label. For example, the first column of GWO-MLP indicates that 89 of the samples with label 1 were predicted correctly, 9 were predicted for label 2, and 48 were predicted for label 3. The data in the lower right corner of each matrix are the overall accuracies of the models.

In terms of classification accuracy, TGAT has the highest overall accuracy of 93.4%, followed by LSTM, and the MLP algorithm optimized by GWO outperforms SVM. Since the samples are divided in chronological order, and at the later stage of the simulation experiments, traffic flows in the road network are accumulating and the intersections are mostly in congested state, the number of samples labelled 3 in the test set is higher, and the number of labels 1 and 4 are relatively low. Traditional models perform poorly in this case of uneven label distribution, such as class 1 labels of GWO-MPL, class 4 labels of LSTM, and class 4 labels of SVM. There will be a situation in which the accuracy of classification of a certain class of labels is less than 60%, whereas the TGAT has an accuracy of more than 86% for all types of labels.

For further comparison, we calculated the Precision, Recall, F1-score, and accuracy, as shown in Table 3, where the unit of measurement is “%”. The chosen metric is the weighted average, which, unlike the macro-average, considers the varying sample counts in each category, thus more accurately reflecting each category’s contribution to the actual metrics, making it more suitable for dealing with the imbalanced dataset in this paper.

As shown in Table 3, as deep learning models, LSTM and TGAT have advantages over traditional machine learning models such as SVM and GWO-MLP across all metrics. TGAT’s Precision metric indicates that for a given category, the average proportion of correctly predicted samples to total predicted samples for that category is 94.01%, while the least effective model, SVM, is only 83.71%. TGAT’s Recall metric indicates that for a given category, the average proportion of correctly predicted samples to actual samples in that category is 93.38%, showing a high coverage of actual samples. The F1-score is the harmonic mean of Precision and Recall. Overall, TGAT achieved the highest values in each metric, making it the most accurate. It should be noted that, as each sample can only belong to one category, accuracy and Recall are equal.

**Figure 6:**
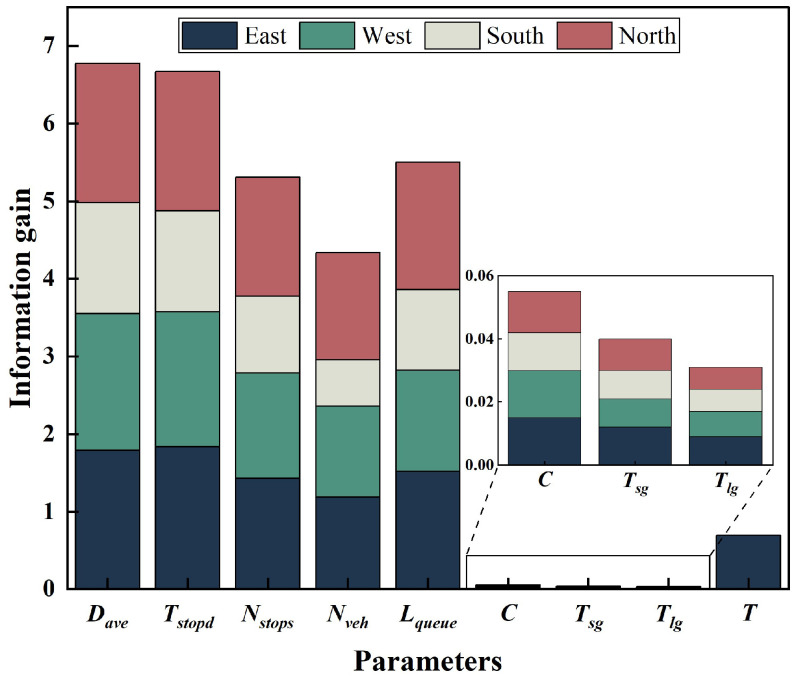
Information gain for each feature.

To verify the role of each feature in classification, the information gain of each feature was calculated according to Formulas (11)–(13). The calculation results are shown in Figure 6.

In the figure, the *x*-axis represents the feature names and the *y*-axis represents the results of information gain, with different colours representing different directions. We aggregate the information gain values for each direction based on the variable names. From the figure, it can be seen that average delay is the most influential feature on traffic state classification, with a total information gain value of 6.776, and its value in each direction exceeds 1.42. Average parking time, average number of stops, and average queue length also play a significant role, with information gain values all above 5.3. The significance of the cycle of the intersection and the average number of vehicles is not high, and the latter exhibits a large variance in information gain values across different directions. The impact of effective green light time and designed traffic capacity is very limited.

### 4.3. Intersection Correlation Results

#### 4.3.1. Interpretability Validation of Correlations

We propose weight evaluation coefficients ω to evaluate the validity of the correlations. Traffic flow is an important parameter for judging the status of an intersection. Usually, the adjacent intersections with larger flow correspond to a larger correlation.

Of all the traffic flows converging on a target intersection, the adjacent intersection corresponding to a particular direction is usually considered to be the most important if it has the largest share of traffic flows. 

Assuming that the set of nearest adjacent intersections of the target intersection i is $N_{i}=\left[I_{1},I_{2},\ldots ,I_{n}\right]$, the set of weights corresponding to each neighbour is $\alpha =\left[\alpha_{1},\alpha_{2},\ldots ,\alpha_{n}\right]$, and the intersection with the largest weight in the above set is $I_{\max}$, with the corresponding weight of $\alpha_{\max}$. We set the weight evaluation coefficient ω, which is calculated by the following formula:

(14) $$\omega =\frac{\sum \alpha_{I_{\max}}}{\beta P_{I_{\max}}}=\frac{\sum \alpha_{I_{\max}}\sum_{j=1}^{n}q_{j}}{\beta \left(q_{S}+q_{L}\right)}$$

where $\alpha_{I_{\max}}$ is the weight of the intersection with the largest weight; $q_{j}$ is the non-right-turning traffic flow from $I_{j}$ to i; $q_{S}$ is the straight traffic flow from $I_{\max}$ to i; $q_{L}$ is the left-turning traffic flow from $I_{\max}$ to i; $P_{I_{\max}}$ is the proportion of the non-right-turning traffic flow from $I_{\max}$ to i among all the non-right-turning traffic flows to i; and β is the adjustment coefficient, which is taken as 1.2 in this paper.

According to the above definition, when ω takes a value close to 1, it means that $\alpha_{i}$ is relatively accurate. It should be noted that since the effect of time on node interaction is considered in TGAT, the same intersection may occur several times.

**Figure 7:**
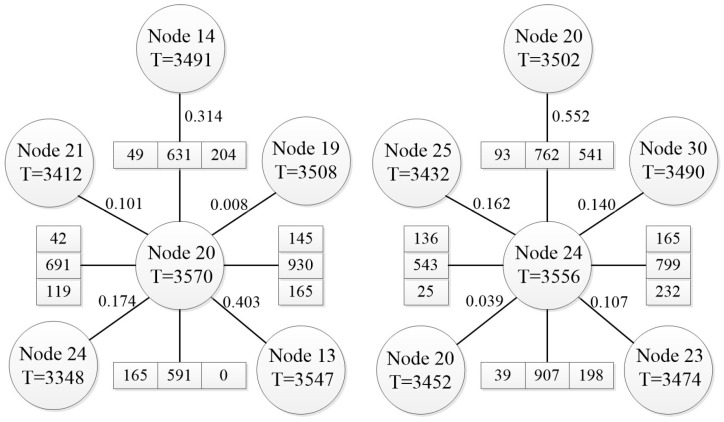
Intersection information.

We describe its computation using the arithmetic example in Figure 7.

In Figure 3, intersection 13 is north of intersection 20. For intersection 20, $\sum \alpha_{I_{\max}}=0.403$, $q_{S}=631$, $q_{L}=204$, and $\sum_{j=1}^{n}q_{j}$ is the sum of flows other than right-turn flows, so ω=1.3751. Similarly for intersection 24 at moment 3556, ω=1.094, where adjacent node 20 appears twice, so $\sum \alpha_{I_{\max}}=0.591$.

In Figure 7, node 20 at time 3570 and node 24 at time 3556 are target nodes, each connected by straight lines to five neighbouring nodes, with the numbers beside the lines representing the weights of the neighbouring nodes. Each target node is connected in the east, west, south, and north directions to an array of three units, representing the traffic flows for left turns, straight-through, and right turns, respectively. For example, at the east approach of intersection 20, the traffic flow is 165 vehicles per hour for left turns, 930 vehicles per hour for straight-through, and 145 vehicles per hour for right turns.

**Figure 8:**
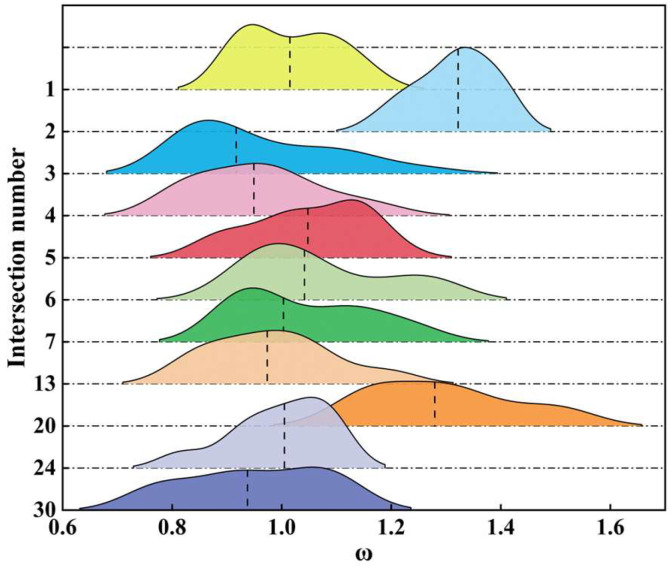
The distribution of ω at intersections on main roads.

For further validation, 11 intersections on two main roads in the road network were selected for the study, with intersections numbered 1–7, 13, 20, 24, and 30, and their ω values were counted and plotted on a ridge diagram, as shown in Figure 8.

The height of the ridge represents the density of the data points in the vicinity of the corresponding ω values, the dotted line in the ridge represents the median, and the horizontal position of the ridge represents the range of ω. As can be seen from Figure 8, the ω values of intersection 2 and intersection 20 are significantly high, most of which are larger than 1.2. Most of the rest of the intersections have weight judgement coefficients around 1, indicating that the correlation of these intersections is closely related to the traffic flow, with the shorter length of the hills at intersections 1, 4, 5, 13, and 24, which indicates that the range of data fluctuation is very small, and the weight judgement coefficients are relatively stable.

#### 4.3.2. Validity Validation of Correlation

In order to verify the validity of the correlation degree more intuitively, the correlation degree calculated by TGAT and the correlation degree calculated by the Whitson model are used as the basis for dividing the road network into different control subareas. Specifically, we divided the 1800–3600 s time period in the experiment into three time periods on average and controlled the input flow in the road network according to 1, 1.2, and 1.5 times that of the actual flow. At the same time, we calculated the correlation values of intersections based on simulation parameters in the second and third time periods and divided the subareas based on this to implement coordinated control. The validity of the correlation is verified by comparing the operational efficiency of the road network as a whole.

**Figure 9:**
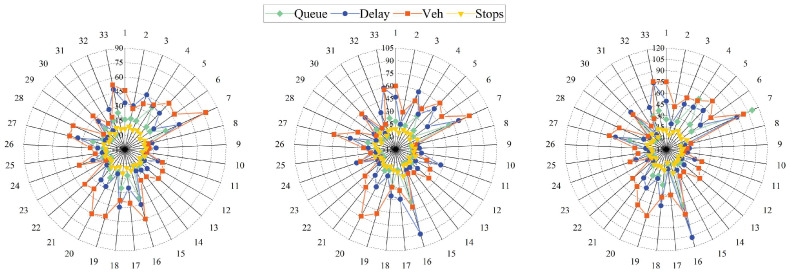
Value of ω at intersections on main roads.

Figure 9 shows the average traffic statistics for each of the 33 intersections without coordinated control for the above three time periods. The three subgraphs from left to right represent the time periods 1800–2400 s, 2400–3000 s, and 3000–3600 s. The data units in the graph are Queue (m), Delay (s), Vehicle (veh/min), and Stops (time/h).

It can be seen that in period 1, the traffic condition is stable, and the average vehicle delay is less than 45 s at most intersections. Only the statuses of intersection 7 and 16 are slow moving as their average queue lengths were longer. In period 2, the degree of congestion increases at intersection 3 and intersection 7, with the average vehicle delay being over 80 s, and all the other data also increase to some extent, with longer average queue lengths and more average stops. In period 3, most of the intersections are already congested, and the average delay of the most serious intersection 16 exceeded 100 s. It is difficult for the coordinated control strategy to be effective in the case of serious congestion on the whole road network, so we only compare the effectiveness of the correlation degree under the coordinated control.

**Figure 10:**
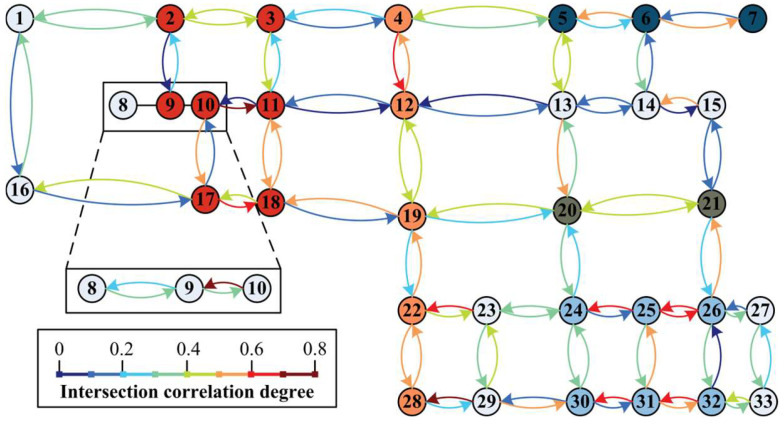
Whitson model correlation in time period 2.

**Figure 11:**
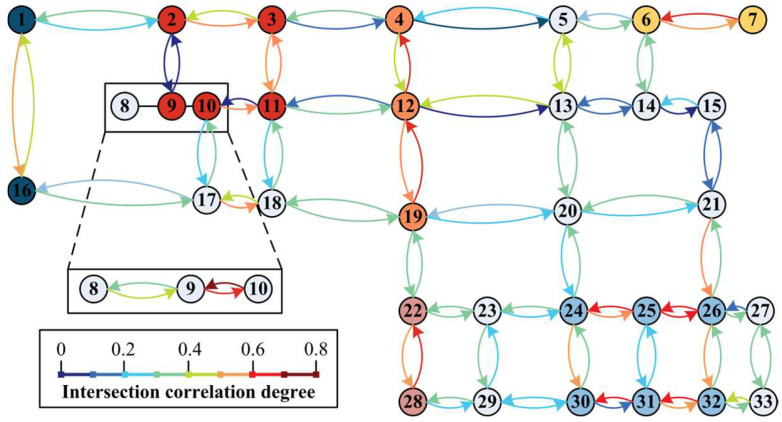
TGAT model correlation in time period 2.

The correlation degree within period 2 is illustrated as an example. According to the traffic data, the correlation degree of each node of the road network can be obtained by bringing it into the Whitson model and the TGAT model, as shown in Figure 10 and Figure 11.

In the Whitson model, it is considered that association is not necessary when the correlation is less than 0.3, and association usually gives better results when it is greater than 0.4. The fuzzy interval is 0.3–0.4. Following this rule, we have filled the nodes within the same subarea with the same colour in Figure 10 and Figure 11, and the unfilled nodes are controlled individually.

**Figure 12:**
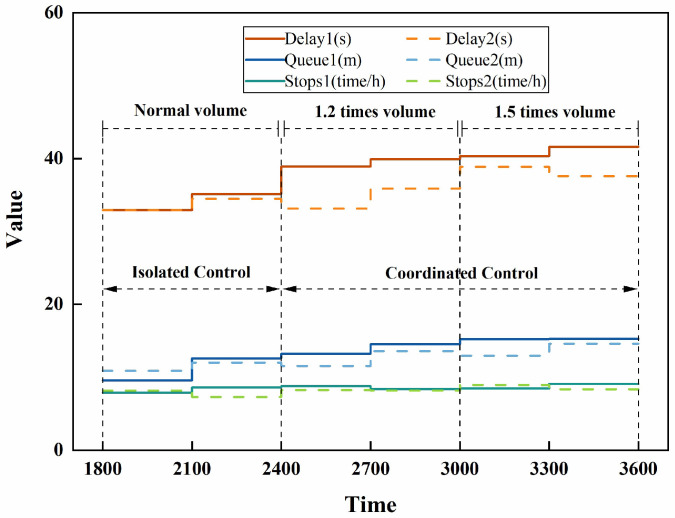
Traffic parameter statistics for different scenarios.

The Whitson correlation-based scenario was labelled as Scenario 1 and TGAT as Scenario 2; the average vehicle delay, average queue length, and average number of stops at all intersections in the road network were counted; and the results are shown in Figure 12.

As can be seen from Figure 12, from 2400 s onwards, with the increase in traffic flow, most traffic indicators show a small increase. The traffic flow in the network is accumulating, according to which the traffic indicators show a gradual increase in general. But the average delay in some periods of Scenario 2 is lower than the previous moment, which indicates that the coordinated control based on the TGAT correlation is more effective. When the traffic volume increases to 1.5 times, the average vehicle delay for Scenario 1 has improved by more than 26% compared to the simulation start time, while the average vehicle delay for Scenario 2 is lower than the value ten minutes prior to Scenario 1, demonstrating that the correlation of the TGAT is still reliable in the presence of a surge in traffic volume. The difference between the two scenarios in terms of average queue length and average number of stops is not significant, mainly because these two values are inherently small, especially since the average number of stops is the value that results from expanding a 5-min statistic to a 1-h one. However, the overall picture is that Scenario 2 is the better option.

## 5. Discussion

1. Integration of entropy and traffic issues

In this paper, the TGAT model has certain flaws, as it cannot quantitatively describe the relationship between various features and classification results. Therefore, we screened out traffic features that significantly impact the intersection category by calculating information gain. In fact, entropy has more connections with traffic issues. Since the types, quantities, and ordering methods of vehicles, even the ordering of traffic signal phases at intersections, are different, if we can consider vehicles and traffic signals as a whole, use entropy to describe their level of disorder, and optimize in the direction of reducing entropy, there might be new solutions to traffic congestion.

2. Intersection state

While the TGAT model does not have the highest classification accuracy for category three, it still has a significant overall accuracy advantage. This is mainly due to limitations in experimental data and computing resources, with less than 5000 samples, which is a relatively small dataset. The model parameters chosen were conservative, unable to fully demonstrate the advantages of TGAT as a deep learning model. The literature [24] has proven that TGAT can handle complex high-dimensional datasets such as Reddit (with 11,000 nodes, 672,447 edges, and 172 dimensions), Wikipedia (with 9227 nodes, 157,474 edges, and 172 dimensions), and Industrial (with 170,843 nodes, 2,135,762 edges, and 100 dimensions). Therefore, it can be anticipated that TGAT’s advantage in classification accuracy will become even more apparent as the data volume increases. In future research, while increasing the number of samples, we can adjust TGAT’s parameters, increase the number of training iterations, reduce the batch size, and try other optimizers such as Adadelta and RMSprop to further enhance the model’s accuracy.

3. Weight evaluation coefficients ω.

We did not choose the intersection on the secondary road to verify ω. On the one hand, we consider that it is a heavy workload. On the other hand, because the traffic flow of the secondary trunk road is relatively small, the traffic flow fluctuates within the data collection interval of every 5 min, which will cause the ω to be unstable.

There is a reason why the ω for intersection 2 and intersection 20 deviate.

(1) Intersection 2 has a difference of more than four times in flow in the east–west and north–south directions and is in close proximity to intersections 8–10. The very high interaction frequency results in large weights, but the actual flow contribution is very low, resulting in a generally large ω.
(2) Intersection 20 has similar causes. Its neighbours in the north–south direction are close, but the contribution of traffic is low, and it is more than 500 m away from the adjacent intersections (13, 19, 21, 24), with a low frequency of interactions, all of which lead to large values of ω.

It can be seen that ω is applicable to non-isolated intersections with relatively uniform traffic distribution on most trunk roads and can be used to judge the validity of the correlation, which is positively correlated with traffic.

## 6. Conclusions and Future Work

Based on the simulation data to extract the intersection traffic characteristics, a TGAT-based intersection correlation model is proposed, which can simultaneously obtain the traffic state of the intersection and the neighbouring intersection correlation. The contributions of this paper lie in the following aspects:

1. The intersection correlation problem is transformed into the neighbour node correlation problem of the nodes in the graph neural network, and the intersection interaction time is defined, the neighbour intersection selection rule is improved, and the traffic state of the intersection can be obtained at the same time.
2. The classification accuracy of the TGAT model proposed in this paper is higher than that of the three classification models, GWO-MLP, LSTM, and SVM, and for the samples with uneven label distribution, the accuracy of each class is higher than 86%, which is a good classification effect and can accurately identify the traffic state of intersections at different moments.
3. We quantitatively calculated the information gain of each traffic characteristic, and the vehicle average delay, average number of stops, and average queue length can significantly affect the classification of intersection status. The effect of traffic capacity and effective green time on classification is smaller, and these features can be appropriately reduced in subsequent research.
4. TGAT-based correlation is positively related to the traffic flow of neighbouring intersections. Compared with the Whitson model, the average vehicle delay, average queue length, and average number of stops of the road network have decreased, which indicates that the correlation degree is effective and can be used as the basis of the traffic control scheme.

There are still some shortcomings in this paper:

1. Due to the limited amount of data, simulation experiments were conducted only on the data of 33 intersections, which should be subsequently expanded to a larger road network environment to verify the efficiency and accuracy of the model in terms of running time.
2. The maximum traffic flow in this paper is 1.5 times that of the investigated flow, and there is no serious congestion in the whole road network. Subsequently, the traffic flow should continue to increase to explore the reliability of the TGAT correlation under oversaturation.
3. Multimodal transportation simulation is very important, and bike lanes are a crucial component of urban road networks [30,31,32,33,34,35]. The experimental area discussed in this paper is located in Beijing, China, where there is a significant presence of non-motorized vehicles that often disrupt motor vehicle traffic. Therefore, when we have more data on bicycles, we can design unique bicycle signal control schemes, which could help improve the efficiency of motor vehicle traffic.

## Figures and Tables

**Table 1:** Intersection characteristic parameters.

No.	Symbol		Meaning
1	Traffic flow characteristics	$D_{ave}$	The average delay of all simulated vehicles in a specified inlet lane (s)
2		$T_{stopd}$	The average stopping time of all simulated vehicles in a specified inlet lane (s)
3		$N_{\mathrm{stops}}$	The average stops of all simulated vehicles in a specified inlet lane (time)
4		$N_{\mathrm{veh}}$	The total number of simulated vehicles in a specified inlet lane (veh)
5		$L_{\mathrm{queue}}$	The average queue length of all simulated vehicles in a specified inlet lane (m)
6	Physical characteristics	C	Total design capacity of intersection inlets (veh/h)
7		$T_{\mathrm{sg}}$	Effective green light time for straight direction (s)
8		$T_{\mathrm{lg}}$	Effective green light time for left-turn direction (s)
9		T	The total signal cycle duration at the intersection (s)

**Table 2:** Parameters for each algorithm.

No.	Name	Parameters
1	TGAT	Number of adjacent nodes (5), learning rate ($3\times 10^{-4}$), dropout (0.15), n_head (3), batch_size (200), n_epoch (500), optimizer (Adam), n_layer (2)
2	GWO-MLP	population size (30), n_epoch (300), implicit layers of the MLP (2), implicit unit number (20), learning rate ($1\times 10^{-4}$)
3	LSTM	network framework (PyTorch), implicit layers (2), implicit unit number (32), dropout (0.15), learning rate ($3\times 10^{-3}$)
4	SVM	kernel function (Radial Basis Function, RBF), n_cross-validation (10)

**Table 3:** Evaluation indicators for each algorithm.

Model	Precision	Recall	F1
GWO-MLP	90.94	90.00	89.33
LSTM	91.73	92.03	91.61
SVM	83.71	82.84	83.00

## Data Availability

Data are contained within the article.
